# Malignant Transformation of Temporal Bone Schneiderian Papilloma Associated with HPV-6

**DOI:** 10.1155/2021/6684254

**Published:** 2021-01-25

**Authors:** O. Marzouk, F. Brasch, I. Todt, P. K. C. Goon, H. Sudhoff

**Affiliations:** ^1^Department of Otorhinolaryngology, Head and Neck Surgery, Medical Faculty OWL, Bielefeld University, Campus Klinikum, Bielefeld, Germany; ^2^Department of Pathology, Academic Teaching Hospital Bielefeld, Bielefeld, Germany; ^3^Department of Dermatology, Peterborough City Hospital, North West Anglia NHS Foundation Trust, Peterborough, UK

## Abstract

**Introduction:**

Temporal bone Schneiderian papillomas (TBSPs) rarely present as a primary tumors arising from the middle ear and mastoid process. The clinical findings and imaging of TBSPs are not specific. Therefore, diagnosis can only reliably be definitively established by histopathology.

**Objective:**

To report a novel case of a malignant transformation of TBSP associated with HPV-6 and to present its management. *Case Report*. A 68-year-old woman presented with conductive hearing loss and recurrent right-sided otorrhoea. Initially, we performed a lateral temporal bone resection and obliteration with abdomen fat. Early histology described TBSP associated with HPV-6. Follow-up detected malignant transformation of the Schneiderian papillomatous variant. Postoperative radiotherapy combined with extended temporal bone resection resulted in a disease-free 17-month period of follow-up. *Discussion*. TBSPs are not very specific, and the diagnosis can only reliably be established by histopathology. There is a risk of malignant transformation, and due to the absence of reliable prognostic markers, strict postoperative follow-up is mandatory and should consist of regular otoscopy, nasal endoscopy, and imaging. This case also supports the importance of extended temporal bone resections as salvage surgery, combining radical surgery with radiotherapy for improved survival rates.

## 1. Introduction

Temporal bone inverted papillomas (Schneiderian-type papillomas, SPs) can rarely present as a primary tumor rising from the middle ear and mastoid process. Additionally, this benign middle ear lesion may originate from the sinonasal tract, accounting for 5% of all tumors [1]. The etiology is unclear, but human papilloma virus (HPV) is considered a risk factor [1, 2]. Inverted papillomas can also occur in the oral cavity, nasopharynx, lacrymal sac, and temporal bone [3–5]. There is a debate in the literature on whether temporal bone Schneiderian papilloma (TBSP) involves the same pathology as sinonasal SP [1, 3]. The involvement of the temporal bone is extremely rare. It can occur either with direct tumor spread via the eustachian tube or originating from ectopic mucosa. Another possible mechanism is related to the iatrogenic implantation of tumor cells during a surgical procedure within the sinonasal tract [2, 6, 7]. A malignant transformation of SPs can occur in about 10% of cases [1, 2]. The typical symptoms of SPs in the temporal bone are otalgia, otorrhoea, and hearing loss [5].

## 2. Case Presentation

**Figure 1:**
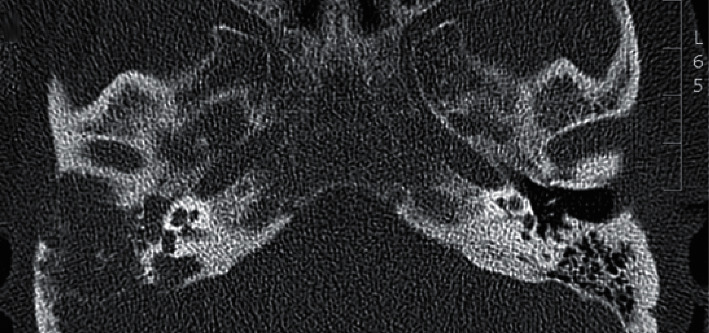
Axial view of the initial temporal CT showed complete opacification of the external auditory canal, middle ear, and mastoid on the right side indicating a soft tissue mass.

**Figure 2:**
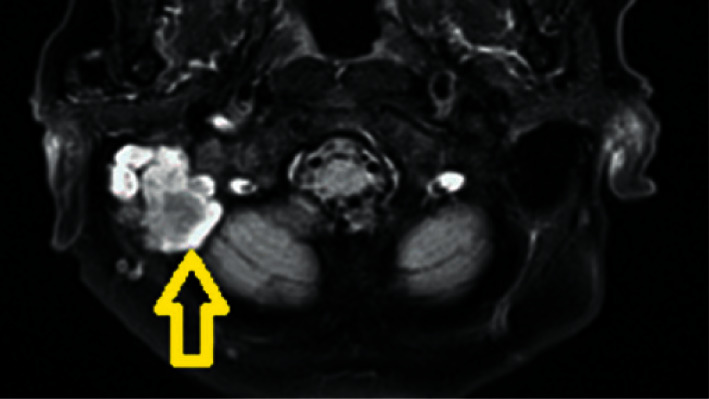
Initial T2-weighted magnetic resonance imaging revealing a mass occupying the right middle ear and mastoid (yellow arrow).

**Figure 3:**
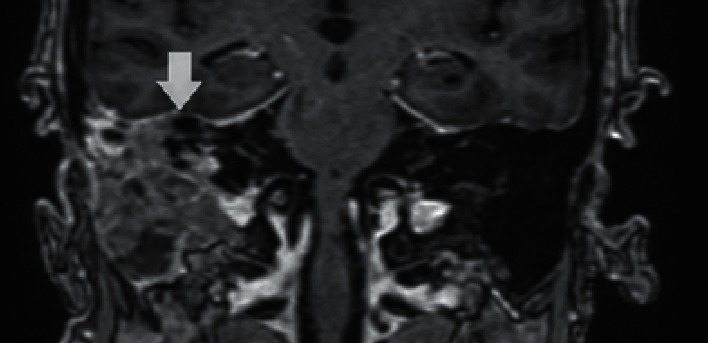
Initial coronal MRI demonstrating a heterogenous tissue mass in the right middle ear mastoid up to the dura of the middle cranial dura (white arrow).

A 68-year-old female Caucasian patient was referred to our clinic for a conductive hearing loss and recurrent right-sided middle ear infections presenting as episodes of painless otorrhoea in January 2019. Otoscopy of the right ear revealed a completely occluded livid-coloured mass with mucopurulent secretions. Anterior rhinoscopy revealed no pathologies. Pure tone audiogram of the right side revealed a moderate to severe mixed hearing loss. There was no medical history of any previous pathology or surgery of the sinonasal tract. An outpatient biopsy of the right external auditory canal revealed an SP. CT scan and magnetic resonance imaging (MRI) of the temporal bone showed a mass that filled the area of the right external auditory canal with penetration into the mastoid cavity with involvement of the dura of the right temporal lobe (Figures 1–3). Subsequently, a combined transmastoidal and transcanal surgical approach of the middle ear was performed, with exenteration of the mastoid cells resulting in a lateral temporal bone resection without any evidence for residual disease after immediate postoperative CT scans. The polypoid tissue found in the external ear canal, middle ear, and mastoid was completely excised in February 2019. Postoperatively, the patient had no facial palsy or vertigo. A temporary otoliquorrhea was successfully managed with conservative therapy. Histopathology diagnosed a Schneiderian-type papilloma with low-risk HPV infection (HPV-6 polymerase chain reaction positive), p16 positive immunohistochemistry without any malignant signs. It exhibited aggregates of hyperplastic respiratory epithelium with invagination throughout the stroma and small cysts in the epithelial lining containing cellular debris and neutrophils. The patient was discharged after 14 days. Subsequently, the patient was monitored by the referring an ENT doctor.

**Figure 4:**
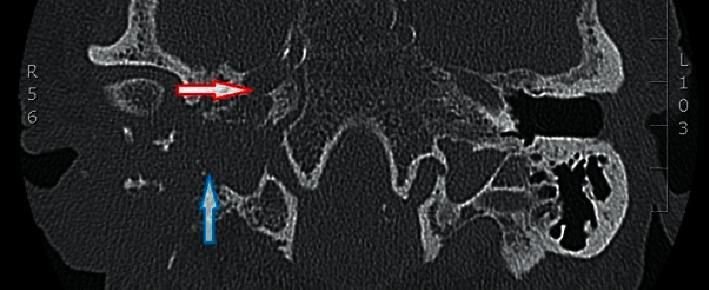
Axial view of temporal CT 5 months after the initial surgery showing complete opacification and massive bone erosion of the middle ear and mastoid on the right side indicating an extended soft tissue mass adjacent to the carotid canal (red arrow) and jugular bulb (blue arrow) and partially obliterating the temporal mandibular joint.

A follow-up CT scan of the temporal bone due to new right-sided facial palsy and complete deafness after 5 months demonstrated a progressive and destructive lesion of the right mastoid and middle ear adjacent to the carotid canal and jugular bulb, erosion of the cochlea, styloid process, and partially involved the temporal mandibular joint in July 2019 (Figure 4).

**Figure 5:**
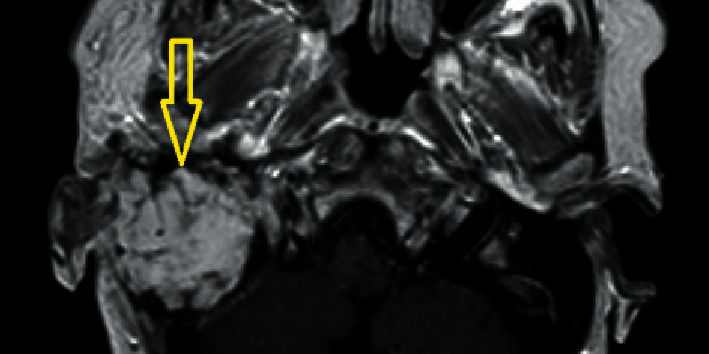
Axial T1-weighted MRI with gadolinium revealed obliteration with abdominal fat (yellow arrow) without any residual tumor 16 months after the extended temporal bone resection.

**Figure 6:**
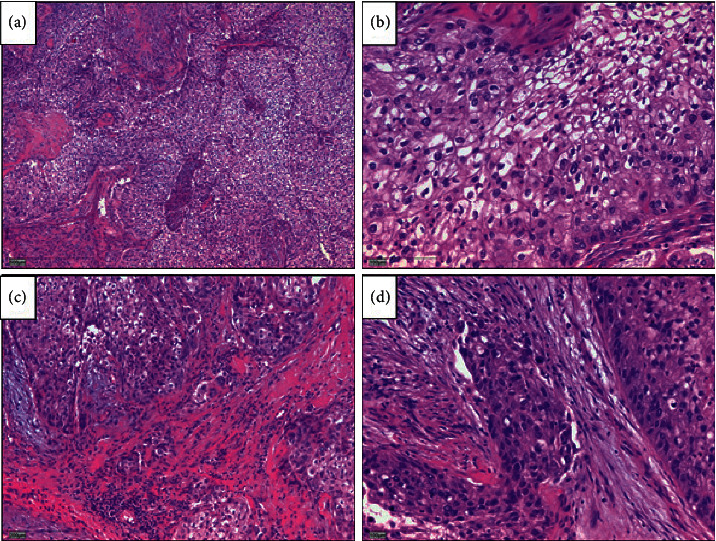
Histopathology revealing parts of the surgical specimen widely occupied by extensions of the temporal bone Schneider papilloma after complete temporal bone resection. Cellular atypia and increased mitoses demonstrate the transition to an invasive, moderately differentiated, nonkeratinizing squamous cell carcinoma.

The patient subsequently underwent a right-sided mastoid exploration, and frozen sections confirmed a squamous cell carcinoma. Subsequently, an extended temporal bone resection with sacrifice of the facial nerve, labyrinth, and cochlea including a duraplasty with abdominal fat obliteration was performed (Figure 5). Pathology reports confirmed a moderately differentiated nonkeratinizing squamous cell carcinoma on a background of SP that had been diagnosed earlier (Figure 6). A postoperative radiotherapy combined with an extended temporal bone resection resulted in a disease-free 17-month follow-up.

## 3. Discussion

To our knowledge, up to the present day, only 33 patients with TBSP have been reported [8]. Of note, in 18 patients, including this current case, the tumor arose as a primary, isolated lesion in the temporal bone, representing a true primary TBSP [8]. In another 14 reported cases, the temporal bone localisation represented an extension from primary sinonasal SP, and in one case, sinonasal involvement is not clearly described [1, 2, 9]. Despite its benign nature, inverted Schneiderian papillomas have a high propensity for local recurrence and may undergo malignant transformation into squamous cell carcinomas [10]. Approximately 20% of these tumors may present with different severities of epithelial dysplasia, which then convey different malignant potentials to the inverted Schneiderian papilloma [11, 12]. The possibility of a coincidence of the simultaneous presence of squamous cell carcinoma and Schneider's papilloma could be excluded by serial sections of the investigated samples. A malignant transformation of the TBSP is exceedingly rare. It has been demonstrated that HPV-6 and 11 (low risk viruses) are the most commonly isolated HPV subtypes in inverted papillomas in the sinonasal tract [13]. On the other hand, HPV-16 and 18 (high risk) are strongly associated with squamous cell carcinoma [14, 15]. Interestingly, our case was associated with HPV-6 but subsequently still demonstrated malignant transformation. HPV-6 and 11 have been rarely associated with malignant transformation in the upper respiratory tract [16–18]. Unusually, the malignant transformation and extensive tumor growth with the destruction of the surrounding structures occurred in a relatively short time from the first surgical intervention in February 2019 to July 2019 along with facial palsy and deafness. So far, the majority of the biological studies have been focused on E6 and E7 from HPV-16 and HPV-18, since they are the most frequent types detected in head and neck cancer. Possibly, an altered immune deviation could have contributed to this rapid malignant transformation [19].

Stone and coworkers reported an inverted papilloma confined to the temporal bone in 1987 [18]. So far, only 7 reported cases have exhibited malignant transformations [8]. Patients with temporal bone inverted papilloma are probably more susceptible to recurrence due to the biology of TBSP. This case highlights that the postoperative follow-up period is crucial, not only to detect recurrences but also to monitor a potential transformation to squamous cell carcinoma [19]. This case also supports the importance of extended temporal bone resections as salvage surgery after preoperative staging and assessment, combining radical surgery with adjuvant radiotherapy for better survival rates [20–22].

## 4. Conclusions

Temporal bone localisation of Schneiderian papilloma may represent a primary tumor or an extension from the sinonasal tract; both are rare. The clinical presentation and radiology of TBSPs are not very specific, and the diagnosis can only reliably be determined by histopathology. There is a risk of malignant transformation, and due to the absence of reliable prognostic markers, strict postoperative follow-up is mandatory and should consist of regular otoscopy, nasal endoscopy, and imaging. Extended temporal bone resection as salvage surgery plus the combination of extended temporal bone resection with radiotherapy seems to improve patient survival.

## Data Availability

The data for this case were accessed through the electronic medical record. They are not available for readers to review as they contain confidential patient health information.
